# Supplementary material for: Vascularization and biocompatibility of poly(ε-caprolactone) fiber mats for rotator cuff tear repair
Source: PLoS One. 2020 Jan 13;15(1):e0227563. doi: 10.1371/journal.pone.0227563 (PMC6957163; doi:10.1371/journal.pone.0227563)
Supplement: Attachment — Submitted filename: Response to Reviewers.docx [file pone.0227563.s002.docx]

MHH – OE7720 – 30625 Hannover

To

The Editors

PLOS ONE

**Zentrum Zahn-, Mund- und Kieferheilkunde**

**Klinik und Poliklinik für Mund-, Kiefer- und Gesichtschirurgie**

**Univ.-Prof. Dr. Dr. N.-C. Gellrich, Direktor**

Dr. Andreas Kampmann

Leiter Forschungslabor MKG-Chirurgie, OE7720

Tel.:	+49 511 532-4841

Mobil:	+49 176 1532-4748

Fax:	+49 511 532-4740

kampmann.andreas@mh-hannover.de

Carl-Neuberg-Straße 1

30625 Hannover

Telefon: 0511 532-0

www.mh-hannover.de

Ihr Zeichen	Ihre Nachricht vom	Unser Zeichen

Dear editor,

First of all, we would like to thank you for the feedback to our manuscript. We appreciate the comments and suggestions on our work and have revised the manuscript accordingly. In addition, we would like to respond to the reviewers’ questions and comments.

Reply to the major comments of reviewer 1:

1. A brief description of the electrospinning parameters has been added to the Materials and methods section of our manuscript. A further description seems not to be necessary as reference 20 is an open access publication. Unfortunately reference 20 was not cited correctly in the reference list, we corrected this, so that the information is available for every reader. (see lines 194-200)
2. We added a brief description of the intravital microscopy data analysis to the Materials and Methods section. (see lines 272-286)
3. Figure 5 J-L are negative controls for the immunhistochemical detection of CD31, we added a short description to the respective paragraph of the results section. (see lines 424 and 425)

Reply to the minor comments of reviewer 1:

1. Thank you very much or this hint. We corrected the description of figure 6 accordingly. (see lines 443, 444 and 446)
2. As described in the Material and methods section, we performed negative controls be omitting the primary antibody, leaving the rest of the used protocol unchanged. This information is already present in the Materials and methods section of the manuscript, but we also added a short description to the respective paragraph of the results section. (see lines 446-448)
3. As suggested by the reviewer we marked the implants in the microscopic pictures presented in Figure 7 using the same scheme as in Figures 3, 5 and 6. (see updated Figure 7)

Reply to the comments of reviewer 2:

1. Our study describes one step in the development process of a new implant for the augmentation of rotator cuff tears. Thereby our research is focused on the vascularization of the implant because this is an important fact, which has considerable impact on the success of the implant. Therefore we don’t agree with the reviewer that our results lack relevance in respect to rotator cuff tears. In different studies an enhanced vascularization was described as beneficial for the healing of rotator cuff tears. In an experimental study Harada et al. [1] used engineered cell sheets for the repair of resected infraspinatus tendons in rats. After 8 weeks the authors found a significant higher ultimate failure load in the cell sheet group compared the contralateral side which served as control without the implantation of a cell sheet. This enhanced mechanical stability was the result of an improved healing process which based on a higher vascularization of the repair site. An enhanced vascularization may also be beneficial for rotator cuff tear repair in human patients. For example Funakoshi et al. [2] showed an increased vascularization of the tendons one or two months after rotator cuff tear repair, which decreased until three months after surgery. In a pilot trial Zumstein et al. [3] used athroscopic repair of rotator cuff tears together with leukocyte- and platelet-rich fibrin. This treatment resulted in a higher vascularization six weeks after repair. Fealy et al. [4] also showed an enhanced vascularization immediately after rotator cuff repair by ultrasound imaging. It has to be mentioned that in both studies vascularization decreased at later time points and there were also no differences in clinical parameters at later time points. Nevertheless it seems to be feasible to assume that an enhanced vascularization is beneficial for the early stages of tendon healing after rotator cuff repair. Therefore we decided to test possible implants for rotator cuff tear repair first in a model that allows the evaluation of vascularization. Implants that were beneficial for vascularization are possible candidates for further research in an animal model of rotator cuff repair. To further enhance our manuscript, we added parts of this argumentation to the discussion section of our manuscript.
2. As suggested by the reviewer we quantified the results in Figure 7 using the software cellSens Dimensions 1.14 (Olympus Deutschland GmbH, Hamburg, Germany). The results of the quantification were added as further information to figure 7. Please refer to our answer to comment 5 for a detailed reply.
3. The different vascularization patterns depicted in Figure 3 can be explained by a different extent of vascular remodeling. The control group showed a rapid vascularization and a high initial functional capillary density beginning with day three. The functional capillary density in the control group showed a decline for the next two days, which was not statistically significant and further declined at the end of the observation period (day 14, statistically significant). A similar vascularization pattern could be observed during the healing of skin wounds. A histological analysis of this process showed an initially high density of small caliber capillaries. With the ongoing healing of the wound the number of vessels declined and vessel caliber increased. [5] We think that a comparable process accounts for the functional capillary density changes in our control group. Therefore we can conclude, that we observed a fast and complete ingrowth of vascularized tissue in the Biomerix scaffold. With respect to the CS-g-PCL group we think that angiogenesis and the growth of the granulation tissue is not that fast as it was observed in the control group and therefore there is still an increase in functional capillary density at the end of the observation period because tissue growth didn’t stopped until that time point.
4. Taken our answer to comment 3 and with respect to the possible clinical application of electrospun fiber mats as scaffolds for the repair of rotator cuff tears we think that the observed changes in functional capillary density are a tolerable, if not desirable feature of such a scaffold. As stated in the answer to comment 1 an enhanced vascularization was described as beneficial for the healing of rotator cuff tears. We therefore think that especially CS-g-PCL fiber mats are well suited for rotator cuff tear repair as they combine a high vascularization with a good biocompatibility.
5. As already stated in our answer to comment 2, we quantified the results of the immunofluorescence detection of macrophages (CD68, CSF1R, CD11b) and T-cells (CD3). Although there were no statistically significant differences for CD11b, the other results supported our hypothesis as they showed a significant lower accumulation of macrophages and T-cells after implantation of PCL and CS-g-PCL implants. We added this new information to the results and discussion section of our manuscript. (see lines 318-324, lines 463-469, lines 481-484, the new Table 4, the new Fig 7 and the related parts of the discussion)
6. We used the Student-Newman-Keuls post-hoc test for differences in means. Once ANOVA gave us statistically significant results, we performed this test to assess which specific pairs of means are different, based on the studenized range distribution. Student-Newman-Keuls is hereby a powerful test and less conservative as other pot hoc tests such as Tukey's range test. We therefore decided to stick to the Student-Newman-Keuls method throughout the whole study.
7. To further enhance the discussion of our manuscript we reorganized the discussion section, thereby adding a discussion of the new information which was added according to the reviewer’s suggestions.

References:

1. Harada Y, Mifune Y, Inui A, Sakata R, Muto T, Takase F, Ueda Y, Kataoka T, Kokubu T, Kuroda R, Kurosaka M. Rotator cuff repair using cell sheets derived from human rotator cuff in a rat model. J Orthop Res. 2017 Feb;35(2):289-296.
2. Funakoshi T, Iwasaki N, Kamishima T, Nishida M, Ito Y, Nishida K, Motomiya M, Suenaga N, Minami A. In vivo vascularity alterations in repaired rotator cuffs determined by contrast-enhanced ultrasound. Am J Sports Med. 2011 Dec;39(12):2640-6.
3. Zumstein MA, Rumian A, Lesbats V, Schaer M, Boileau P. Increased vascularization during early healing after biologic augmentation in repair of chronic rotator cuff tears using autologous leukocyte- and platelet-rich fibrin (L-PRF): a prospective randomized controlled pilot trial. J Shoulder Elbow Surg. 2014 Jan;23(1):3-12.
4. Fealy S, Adler RS, Drakos MC, Kelly AM, Allen AA, Cordasco FA, Warren RF, O'Brien SJ. Patterns of vascular and anatomical response after rotator cuff repair. Am J Sports Med. 2006 Jan;34(1):120-7.
5. Johnson KE, Wilgus TA. Vascular Endothelial Growth Factor and Angiogenesis in the Regulation of Cutaneous Wound Repair. Adv Wound Care (New Rochelle). 2014 Oct 1;3(10):647-661.

We thank the reviewer for the careful review of our manuscript and the valuable suggestions. We also corrected some minor mistakes and language inaccuracies and added the missing information according to the ARRIVE guidelines. We feel that the changes made according to their suggestions have markedly improved the quality of the manuscript, and would be happy if it now met the criteria for publication in PLOS ONE.

Sincerely,
