# Supplementary material for: Vascularization and biocompatibility of poly(ε-caprolactone) fiber mats for rotator cuff tear repair
Source: PLoS One. 2020 Jan 13;15(1):e0227563. doi: 10.1371/journal.pone.0227563 (PMC6957163; doi:10.1371/journal.pone.0227563)
Supplement: Attachment — Submitted filename: Response to Reviewers_2.docx [file pone.0227563.s003.docx]

MHH – OE7720 – 30625 Hannover

To

The Editors

PLOS ONE

**Zentrum Zahn-, Mund- und Kieferheilkunde**

**Klinik und Poliklinik für Mund-, Kiefer- und Gesichtschirurgie**

**Univ.-Prof. Dr. Dr. N.-C. Gellrich, Direktor**

Dr. Andreas Kampmann

Leiter Forschungslabor MKG-Chirurgie, OE7720

Tel.:	+49 511 532-4841

Mobil:	+49 176 1532-4748

Fax:	+49 511 532-4740

kampmann.andreas@mh-hannover.de

Carl-Neuberg-Straße 1

30625 Hannover

Telefon: 0511 532-0

www.mh-hannover.de

Ihr Zeichen	Ihre Nachricht vom	Unser Zeichen

Dear editor,

First of all, we would like to thank you again for the feedback to our manuscript. We appreciate the fair and constructive comments provided by the reviewer. In the following text, please find our point-by-point reply.

Reply to the minor comments of reviewer 3:

1. We thank the reviewer for this suggestion. To better illustrate the reasons why electrospun PCL is well suited for rotator cuff tear repair und to show why we used the specific configuration of the PCL fiber mats, we added additional information, concerning the use of PCL in different tissue engineering application and especially for the repair of rotator cuff tears. Implants for rotator cuff tear repair should have a positive impact on tendon healing by supporting cellular infiltration and guiding the regeneration of an organized tendon structure. By fabrication of fiber mats with directed or undirected fiber orientation they can be adopted to the microarchitecture of the different sections of the native tendon. The specific design of the fiber mat in turn stimulates the regeneration of organized tendon structures. In a previous study we analyzed electrospun PCL fiber mats with undirected fiber orientation, that were intended to mimic the specific fiber orientation at the tendon-bone transition in the femur chamber in rats [Gniesmer et al. 2019]. The current study was designed to examine fiber mats with directed fiber structures, which simulate the tendon-muscle transition. These electrospun PCL fiber mats are intended to form the lead structure for the transition zone of the rotator cuff. (see lines 180-195)
2. We apologies for the confusion caused. The expression “ingrowth of capillaries” was used in the abstract of the manuscript and the conclusion at the end of the discussion section. For a clear discussion of our results we changed the expression to “formation of vascularized tissue and the ingrowth of cells” (see lines 137 and 624)
3. We updated the Materials and methods section and added a brief description how the evaluation of porosity and pore diameter was done. (see lines 227-233)
4. The reviewer is right at this point; by mistake we omitted essential information about the preparation of the observation window. We corrected this mistake in the revised version of the manuscript. (see lines 270 and 271)
5. In the revised version of the manuscript we tried to harmonize the number of digits after the decimal point where possible. In Table 1, 2 and 3 we used one decimal place. In Table 4 the use of three decimal places was necessary and justified by our data. We also corrected wrong use of decimal point and decimal comma in all tables. (see Table 1, 3 and 4)

Reference:

Gniesmer S, Brehm R, Hoffmann A, de Cassan D, Menzel H, Hoheisel A-L, et al. In vivo analysis of vascularization and biocompatibility of electrospun polycaprolactone fiber mats in the rat femur chamber. J Tissue Eng Regen Med. 2019 Apr 26;0(ja). https://doi.org/10.1002/term.2868

We thank the reviewer for the careful review of our manuscript and the valuable suggestions, which helped to further improve the quality of the manuscript and would be happy if it now met the criteria for publication in PLOS ONE.

Sincerely,
